# Impact of Composition Ratio on the Expansion Behavior of Polyurethane Grout

**DOI:** 10.3390/ma17081835

**Published:** 2024-04-16

**Authors:** Xiaolong Li, Cen Peng, Yanna Ao, Meimei Hao, Yanhui Zhong, Bei Zhang

**Affiliations:** 1 School of Water Conservancy and Transportation, Zhengzhou University, Zhengzhou 450001, China; 2 National Local Joint Engineering Laboratory of Major Infrastructure Testing and Rehabilitation Technology, Zhengzhou 450001, China

**Keywords:** self-expanding polymers, expansion characteristics, ingredient proportion, simulation study

## Abstract

Different formulations of foaming polyurethane grout offer controlled expansion rates. This is crucial for precision in filling voids without exerting excessive pressure on surrounding structures, which could potentially cause damage. This study focuses on the impact of composition on the expansion performance of tailor-made polyurethane grouting materials. Initially, multiple unknown chemical reaction kinetic parameters were identified by combining free expansion tests, which involved measuring density and temperature changes, with the particle swarm optimization algorithm. A numerical simulation, integrating chemical kinetic models and fluid flow equations, was established to replicate the free expansion process of polyurethane grout in a cup, aligning with our experimental results. Subsequently, we analyzed the polymerization process of polyurethane grout with varying compositions to determine the effect of composition ratios on grout expansion. Our findings reveal that the expansion ratio of foaming polyurethane is predominantly influenced by the concentrations of physical and chemical foaming agents, followed by isocyanate concentration. Polyol, in contrast, exerts a relatively minor influence. Furthermore, the solubility of the physical foaming agent in the grout determines both its maximum allowable concentration and its maximum contribution to volume increase. This study provides valuable insights for the design and selection of polyurethane grout components tailored to diverse applications.

## 1. Introduction

Customized polyurethane grouting materials with varying expansion ratios have been widely developed for infrastructure rehabilitation, including applications in road maintenance, slab lifting, structure reinforcement, and underground anti-seepage [1,2,3,4]. The fundamental principle of polyurethane grouting lies in injecting a two-component polyurethane grout into specified locations, aimed at filling voids and cracks, waterproofing, and enhancing load-bearing capacity. This process generates an expansion force that acts upon either the surrounding geological or environmental medium or the grout itself, facilitating the desired structural adjustments or repairs.

Different formulations of foaming polyurethane grout lead to different expansion ratios. It is essential to achieve the appropriate expansion ratio to ensure that the grout effectively fills voids without exerting excessive pressure on the surrounding structure, which could result in insufficient filling and potential damage to the original structure. Therefore, it is crucial to adjust the grout components according to the specific requirements for expansion performance in practical projects.

Numerical simulation serves as a crucial method for investigating expanding polyurethane foams. Typically, the polymer grout is regarded as a generalized Newtonian fluid, with the expansion process simulated as a fluid flow operation. Researchers have employed macro-scale computational fluid dynamics (CFD) and multi-scale modeling to forecast the properties of polyurethane foam [5,6,7]. These methods connect large-scale fluid behavior with the minute interactions of bubbles. Despite these advances, the nature of foaming is complex, involving the interplay of liquid and gas, intricate chemical reactions, and varied thermal processes, like heat transfer and phase changes. The simulation requires careful consideration of many factors, such as the pre-exponential factor and activation energy parameters in kinetic equations, as well as parameters in solubility equations, each contributing to the complexity of the model. Researchers, such as Gerier [8], Lipshitz [9], Bouayad et al. [10], and Dimier [11], have conducted extensive experiments and thermal analyses to identify these parameters. To streamline this process, Raimbault [12] devised an analytical model that simplifies the identification of key parameters for curing kinetics and viscosity. Similarly, Jia et al. [13] refined the parameters for the solubility model of the physical blowing agent, applying mass conservation and the Clapeyron equations. Abdessalam [14] took a different approach with an inverse identification method, which integrates data from dynamic rotational rhinometry and FOAMAT system tests with foaming simulations using the finite pointset method. This strategy aims to lessen the extensive labor involved in measuring numerous parameters that vary with different formulations.

In the field of grouting engineering, the expansion characteristics have a significant impact on the mechanical properties. Yang et al. [15] utilized microcellular foaming technology, employing CO_2_ and N_2_ as co-foaming agents, to modulate the shrinkage behavior of hexamethylene diisocyanate (HDI)-based thermoplastic polyurethane (TPU) foam. They examined the influence of shrinkage rate, expansion rate, and cell size on the mechanical properties of the foam and found that the mechanical properties of TPU foams with a smaller shrinkage ratio are much higher than those with a larger initial expansion ratio and a similar final expansion ratio. Vipulanandan C et al. [16] investigated the curing process of hydrophilic polyurethane, focusing on how varying water–grouting ratios influence volume change. Their findings indicate a direct correlation between these ratios and the observed increases in pressure and temperature at peak curing. Sabri, M.M. et al. [17] manipulated the volume expansion ratio of expandable polyurethane resin by adjusting the injected resin amount and examining the mechanical properties that ensured. This work culminated in establishing a stress–strain diagram at varying densities and expansion ratios.

Researchers have conducted investigations into the impact of certain components on both the foaming process of polyurethane and its mechanical properties. Zhuang et al. [18] observed that incorporating a chain extender into the TPU matrix enhances the branching of the molecular chain, resulting in an expansion rate and compressive strength of composite foam that are two to threefold that of the unmodified sample. Lai et al. [19] studied the effects of varying chain extender levels on the mechanical and foaming properties of thermoplastic polyurethane materials. Oppon et al. [20] explored the effect of preheating temperature on foaming duration and expansion rate, discovering that higher preheating temperatures reduce the time required for foaming and accelerate the expansion rate. Karimi et al. [21,22] used the population balance equation to assess how different types and quantities of the physical blowing agent, along with water content, affect the density and temperature of polyurethane materials. Nofar et al. [23] extensively analyzed how the content of hard segments influences the foaming behavior of thermoplastic polyurethane, revealing that an increase in hard segment crystallization during the saturation process restricts foam expansion.

Despite extensive research on the chemical kinetic parameters and numerical simulations of the polyurethane foam filling process for mold filling, studies on polyurethane grouting have predominantly focused on the diffusion and mechanical properties of polyurethane grout with set components. The complexity of the formula and the operational environment introduce uncertainties in the numerical model of expansion behavior in polyurethane grouting projects. Currently, optimizing grout composition for desired expansion behavior largely depends on existing experience or experimental methods, which are costly and inefficient. Consequently, a significant challenge remains in developing a numerical method of grout expansion that can accommodate polyurethane grouting with undetermined kinetic parameters.

In this study, we conducted an investigation into the quantitative effect of composition ratios on the expansion properties of polyurethane grouting materials through numerical simulation. Initially, a free expansion test of the grout was carried out to monitor changes in grout density and temperature over time. Subsequently, utilizing particle swarm optimization (PSO), we identified the chemical reaction kinetics parameters of the grouting materials and established material models for polyurethane. Building upon this foundation, we developed a numerical simulation to predict the expansion ratio and density of polyurethane grouting materials. Furthermore, an analysis was performed to assess the impact of different composition ratios, including isocyanate and polyol, as well as physical and chemical blowing agents, on expansion performance. This study serves as a fundamental step towards designing formulas for polyurethane grouting materials with desired expansion properties.

## 2. Chemical Kinetic Parameter Identification

**Figure 1:**
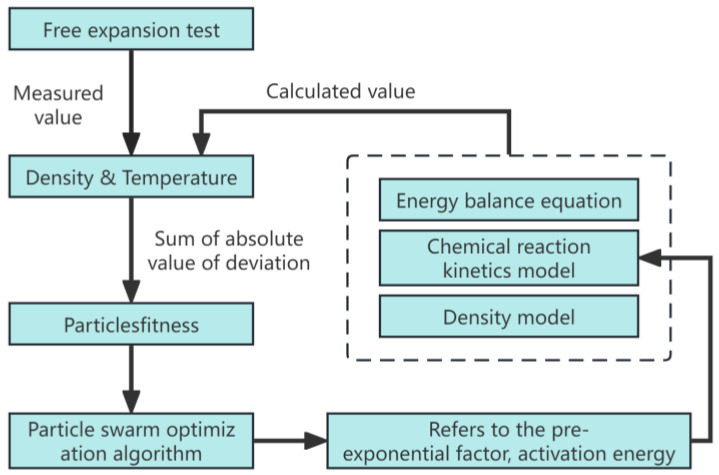
Flow diagram of the particle swarm algorithm.

The pre-exponential factor and activation energy are crucial parameters in the chemical reaction kinetics equation of polymer grouting materials. These kinetic parameters are determined using a particle swarm inversion algorithm, which combines an energy conservation equation, chemical reaction kinetic model, and density model. Through continuous optimization of particle swarm fitness, we were able to ascertain the pre-exponential factor and activation energy for our custom polymer grouting material through progressive refinement. The optimization process concludes when the discrepancies in density and temperature between calculated and experimental values reach their minimum. These results provide a solid foundation for the numerical simulation of the polymer expansion process in subsequent steps. Figure 1 presents a schematic diagram of our research, illustrating the application of the particle swarm inversion algorithm more clearly.

### 2.1. Preparation of the Polyurethane Grouting Material

In this study, a new type of polyurethane polymer grout was developed, which enhanced the identification of the chemical kinetic parameters through more obvious foaming properties, and we established an accurate numerical model of the expansion process of polyurethane grout. Through a series of experiments and comparative evaluations, optimal raw materials and their proportions were identified. The primary components for crafting this polyurethane grout encompass isocyanates, polyols, catalysts, blowing agents, foam stabilizers, and a selection of additional additives.

**Figure 2:**
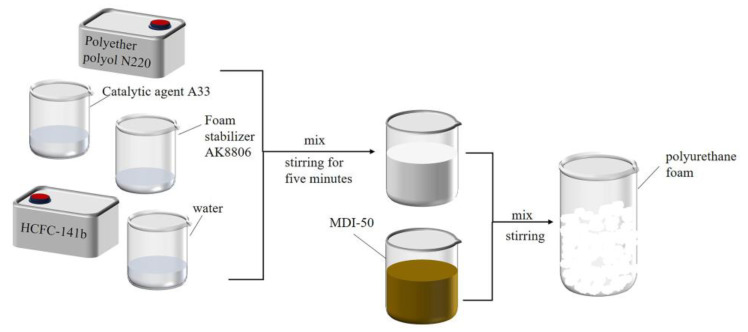
Schematic diagram of preparing polyurethane grout.

Table 1 presents the selected raw materials and their precise ratios used to create the polymer grout via a one-step method [24]. To prevent the material from sticking to the container post-reaction, the inner surface of the container was lightly coated with lubricating oil before beginning the experiment. At room temperature, the polyether polyol, foaming agent, foam stabilizer, and catalyst were combined in the container according to the specified proportions. Once thoroughly mixed, MDI-50 was quickly integrated, ensuring rapid reaction and expansion to produce the polyurethane grout. The entire process was illustrated in Figure 2.

### 2.2. Free Expansion Test

**Figure 3:**
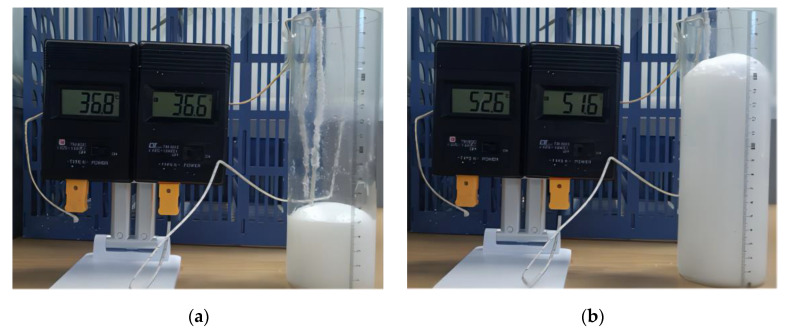
Volume and temperature measurement during grout expansion: (**a**) t = 14 s; (**b**) t = 28 s.

As shown in Figure 3 using a 62 mm diameter cylinder marked with a scale, we added the reactive raw materials in specified ratios at room temperature to conduct the free expansion experiment. The initial height of polyurethane mixture in the cylinder is represented by $h_{0}$. During the expansion, we continuously measured and recorded the height and temperature of the grout, enabling us to calculate the volume expansion ratio and density changes. The shape of the grouting materials undergoes changes over time, with the volume initially approximated as a cylinder during the early stage of expansion. As the grouting materials expands to a certain extent, its outline becomes more prominent, and the upper convex part can be approximated as hemispherical [25]. Therefore, the volume expansion ratio of the grouting materials in both stages is as follows:

(1) $$\varphi =\left\{\begin{array}{c} \frac{\pi r^{2}h_{1}(t)}{\pi r^{2}h_{0}}\\ \frac{\pi r^{2}h_{1}(t)+\frac{2}{3}\pi r^{2}[h(t)-h_{1}(t)]}{\pi r^{2}h_{0}} \end{array}\right.$$

where r is radius of the cylinder, h(t) is the height from the bottom of the container to the highest point of the grouting at time t, and $h_{1}(t)$ is the height of the grouting cylinder at time t.

The density of the grout at different times is determined by dividing its mass by the volume of expansion. Based on the energy balance equation, this experimental data lays the foundational groundwork for the subsequent inversion of the kinetic parameters of the polyurethane grout.

### 2.3. Chemical Reaction Kinetics Model

The expansion process of polyurethane related to chemical reactions is complex, involving several chemical reactions and intermediate products. It primarily encompasses two key chain growth reactions: the gelation reaction between isocyanate and hydroxyl groups, and the foaming reaction where isocyanate interacts with water. The chemical reaction can be represented as follows:

(2) $$R-NCO+R\prime -OH\to R-NH-\overset{O}{\overset{∥}{C}}-O-R\prime$$

(3) $$2R-NCO+H_{2}O\to R-NH-\overset{O}{\overset{∥}{C}}-\mathrm{NH}-R+CO_{2}↑$$

To understand the foaming mechanism of polyurethane grout, it is essential to analyze the conversion rate and residual concentrations of reactants over time. This analysis enables the correlation of density, viscosity, and other physical parameters with the reactant concentrations. Developing a detailed kinetic model of the polyurethane grout is, therefore, crucial, with a focus on key parameters, such as pre-factor and activation energy. The reaction rates for both gelation and foaming are effectively determined by the changing concentrations of hydroxyl groups and water. Based on the principles of the Arrhenius equation, the kinetic equations for these reactions in the polymer paste are as follows [26,27]:

(4) $$\frac{dX_{\mathrm{OH}}}{dt}=A_{\mathrm{OH}}\cdot \exp(-\frac{E_{\mathrm{OH}}}{RT})\cdot C_{\mathrm{OH}}^{0}\cdot (1-X_{\mathrm{OH}})\cdot (\frac{C_{\mathrm{NCO}}^{0}}{C_{\mathrm{OH}}^{0}}-2\frac{C_{W}^{0}}{C_{\mathrm{OH}}^{0}}X_{W}-X_{\mathrm{OH}})$$

(5) $$\frac{dX_{W}}{dt}=A_{W}\cdot \exp(-\frac{E_{W}}{RT})\cdot C_{\mathrm{OH}}^{0}\cdot (1-X_{W})\cdot (\frac{C_{\mathrm{NCO}}^{0}}{C_{\mathrm{OH}}^{0}}-2\frac{C_{W}^{0}}{C_{\mathrm{OH}}^{0}}X_{W}-X_{\mathrm{OH}})$$

where $X_{\mathrm{OH}}$ is the conversion rate of the hydroxyl component. $A_{\mathrm{OH}}$ is the pre-exponential factor of the gel reaction, m^3^/g equiv/s. $E_{\mathrm{OH}}$ is the activation energy of the gel reaction, J/g mol. R is the ideal gas constant. T is the current temperature, K. $C_{{\;\;}_{\mathrm{OH}}}^{0}$ is the molar concentration of the hydroxyl component at the initial time, mol/m^3^. $C_{{\;\;}_{\mathrm{NCO}}}^{0}$ is the molar concentration of the isocyanate component at the initial time, mol/m^3^. $C_{{\;\;}_{W}}^{0}$ is the initial molar concentration of the water component, mol/m^3^. $X_{W}$ is the conversion rate of the water component. $A_{W}$ is the pre-exponential factor of the foaming reaction, m^3^/g equiv/s. $E_{W}$ is the activation energy of the foaming reaction, J/g mol.

For different chemical reactions, such as the gel reaction and foaming reaction of polymer grout, as well as polymer grout of different components, the pre-factor and activation energy are different, which usually means that the pre-factor and activation energy are calculated and solved by experiments [8]. In this paper, the inversion method was adopted to identify the pre-factor and activation energy of self-made polymer grouting materials. Based on the measured temperature and density data, the method used a particle swarm optimization algorithm to invert the chemical reaction kinetic parameters of polymer grouting, with fewer tests and high accuracy.

### 2.4. Energy Balance Equation

Both the gelation reaction and foaming reaction are exothermic reactions, while the physical foaming agent absorbs heat due to evaporation. The energy balance equation of expanding polyurethane grout under adiabatic condition is as follows [28]:

(6) $$\left[C_{P}+r_{{\mathrm{CO}}_{2}}C_{{\mathrm{CO}}_{2}}+r_{W}C_{W}+r_{\mathrm{BG}}C_{\mathrm{BG}}+r_{\mathrm{BL}}C_{\mathrm{BL}}\right]\frac{\mathrm{dT}}{\mathrm{dt}}=\left[\frac{{\left(-\Delta H\right)}_{\mathrm{OH}}C_{\mathrm{OH},0}}{\rho_{P}}\right]\frac{dX_{\mathrm{OH}}}{\mathrm{dt}}+\left[\frac{{\left(-\Delta H\right)}_{W}C_{W,0}}{\rho_{P}}\right]\frac{dX_{W}}{\mathrm{dt}}-\lambda \left(-\frac{dr_{\mathrm{BL}}}{\mathrm{dt}}\right)$$

where $C_{P}$ is the heat capacity of polymer grouting, $C_{{\mathrm{CO}}_{2}}$ is the heat capacity of carbon dioxide, $C_{W}$ is the heat capacity of water, $C_{\mathrm{BG}}$ is the heat capacity of the gaseous physical foaming agent, $C_{\mathrm{BL}}$ is the heat capacity of the liquid physical foaming agent, $r_{{\mathrm{CO}}_{2}}$ is the mass fraction of carbon dioxide, $r_{W}$ is the mass fraction of water, $r_{\mathrm{BG}}$ is the mass fraction of the gaseous physical foaming agent, $\Delta H_{W}$ is the heat of the foaming reaction, and λ is the heat of the evaporation and absorption of the physical foaming agent.

### 2.5. Material Properties of Polyurethane

(1) Density model

Polyurethane grout can be classified as a macroscopically homogeneous liquid, encompassing two distinct phases: a liquid phase and a gas phase. The gaseous components primarily originate from the evaporation of the physical blowing agent and the generation of carbon dioxide, factors which significantly contribute to alterations in density. The liquid phase comprises both the reactants and the unreacted substances, such as polyurethane and the liquid physical foaming agent that is dissolved in the mixture. According to references [6,29], the density of this system is expressed as follows:

(7) $$\rho =\frac{1+r_{W}^{0}+r_{\mathrm{BL}}^{0}}{X_{W}\frac{R_{g}Tr_{W}^{0}}{PM_{W}}+\frac{r_{\mathrm{BG}}RT}{PM_{B}}+\frac{r_{\mathrm{BL}}}{\rho_{\mathrm{BL}}}+\frac{r_{W}}{\rho_{W}}+\frac{1}{\rho_{P}}}$$

where $r_{{\;\;}_{W}}^{0}$ is the initial mass fraction of water, $r_{{\;\;}_{\mathrm{BL}}}^{0}$ is the initial mass fraction of physical foaming agent, $R_{g}$ is the ideal gas constant, $r_{\mathrm{BG}}$ is the mass fraction of the gaseous physical foaming agent, $r_{\mathrm{BL}}$ is the mass fraction of the liquid physical foaming agent, $r_{W}$ is the mass fraction of water, $M_{W}$ is the molar mass of water, $M_{B}$ is the molar mass of the physical foaming agent, $\rho_{\mathrm{BL}}$ is the density of the liquid physical foaming agent, $\rho_{W}$ is the density of water, and $\rho_{P}$ is the density of the polymer mixture.

(2) Viscosity model

The viscosity change in polymer grouting can usually be described by the Castro–Macosko model [30], as follows:

(8) $$\mu_{f}(T,X_{\mathrm{NCO}})=\mu_{\infty }\exp(\frac{E_{\mu }}{RT})\cdot {\left(\frac{X_{\mathrm{NCO},\mathrm{gel}}}{X_{\mathrm{NCO},\mathrm{gel}}-X_{\mathrm{NCO}}}\right)}^{\left(a+bX_{\mathrm{NCO}}+cX_{\mathrm{NCO}}^{2}\right)}$$

where $X_{\mathrm{NCO},\mathrm{gel}}$ is the conversion rate of isocyanate gel, $\mu_{\infty }$ and $\frac{E_{\mu }}{R}$ are the coefficients, and a, b, and c are the viscosity model coefficients, with values of 1.5, 1, and 0, respectively.

(3) Thermal conductivity

The equation for the thermal conductivity uses an empirical density-dependent expression obtained by Marciano based on Harper’s test [31,32]. The thermal conductivity formula is as follows:

(9) $$\lambda_{F}=\left\{\begin{array}{c} 8.7006\times 10^{-8}\rho_{F}^{2}+8.4674\times 10^{-5}\rho_{F}+1.16\times 10^{-2}\;\;\;\;\rho_{F}\geq 48{\mathrm{kg}/m}^{3}\\ 9.3738\times 10^{-6}\rho_{F}^{2}-7.3511\times 10^{-4}\rho_{F}+2.956\times 10^{-2}\;\;\;\;\rho_{F}<48{\mathrm{kg}/m}^{3} \end{array}\right.$$

where $\lambda_{F}$ is the thermal conductivity of the grouting, and $\rho_{F}$ is the density of the grouting.

(4) Solubility determination of HCFC-141b

**Figure 4:**
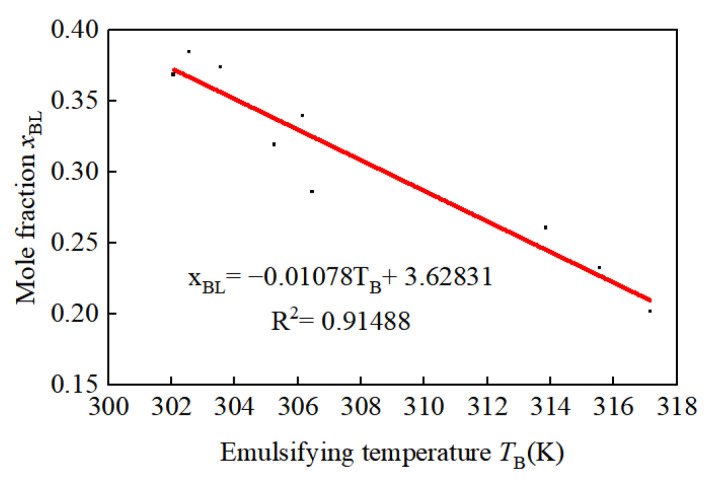
The relationship between HCFC-141b mole fraction and emulsification temperature.

Following the methodology outlined by S.A. Baser [26], experiments were conducted to assess the emulsification temperature of the physical foaming agent at varying molar fractions, the results of which are illustrated in Figure 4. A linear relationship between the molar fraction $x_{\mathrm{BL}}$ of HCFC-141b within the mixture and the emulsification temperature $T_{B}$ was established through fitting. The linear correlation coefficient was found to be 0.91488. The linear correlation coefficient between the variables in the fitting function indicates a strong fit. By utilizing the first-order function of the mole fraction in relation to the emulsification temperature, we can further elucidate the solubility model of HCFC-141b, as follows:

(10) $$\frac{dr_{\mathrm{BL}}}{dt}=\left\{\begin{array}{c} \frac{116.9}{700}\cdot \frac{-0.01078}{{\left(0.01078T-2.62831\right)}^{2}}\cdot \frac{dT}{dt}\;\;\;T\geq T_{\mathrm{BL},0}\\ 0\;\;\;\;\;\;\;\;\;\;\;\;\;\;T<T_{\mathrm{BL},0} \end{array}\right.$$

### 2.6. Identification of Pre-Exponential Factor and Activation Energy

The particle swarm optimization (PSO) algorithm, initially introduced by Kennedy and Eberhart, is a stochastic search algorithm renowned for its straightforward implementation and robust global search capability [33]. Utilizing this algorithm, based on the measured temperature and density data, the pre-exponential factor and activation energy within the kinetic equation of polymer grouting material’s chemical reaction are identified. The procedural steps are as follows:

(1) Define the population size n, the particle search space range and dimension d, learning factors $c_{1}$ and $c_{2}$, the iteration count t, and the convergence accuracy ε. The search space encompasses the pre-exponential factor and activation energy.
(2) Initialize the position and velocity of each particle in the population. Positions are randomly set within the estimated ranges for the pre-exponential factor and activation energy, expressed as *x_i_^t^* = (*A*_OH_, *E*_OH_, *A*_W_, *E*_W_), *i* = 1: *n*. Particle velocities are also randomly generated in the form of *v_i_^t^* = (*v_i_*_1_, *v_i_*_2_, *v_i_*_3_, *v_i_*_4_), *i* = 1: *n*, applicable to each particle.
(3) Initialize the individual and group historical optimal values for each particle. Initial parameters for the polyurethane grouting material, such as the initial concentration of the hydroxyl $C_{{\;\;}_{\mathrm{OH}}}^{0}$, isocyanate $C_{{\;\;}_{\mathrm{NCO}}}^{0}$, and water component $C_{{\;\;}_{W}}^{0}$, the initial mass fraction of the physical foaming agent $r_{{\;\;}_{\mathrm{BL}}}^{0}$, the initial density of the grout $\rho_{{\;\;}_{P}}^{0}$ and its initial temperature $T_{0}$, are inputted. In the process of steps (1)–(3) above, the parameter values that need to be entered and set are shown in Table 2.

The forward modeling serves as a key tool for resolving the chemical reaction kinetics and heat balance equations of polyurethane grout. Through this approach, we obtain time-dependent conversion rate of grout components and the variations in temperature at different times. The density model is then employed to calculate the grout density. To evaluate the fitness of the particles fit, we use the sum of the absolute deviations between the calculated and measured values of grout temperature and density at various time points. The formula for this calculation is as follows [34]:

(11) $$f_{i}^{t}=\sum_{k=1}^{m}\left|T_{k}-T_{k}^{\prime }\right|+\sum_{j=1}^{l}\left|D_{j}-D_{j}^{\prime }\right|$$

where *T*_k_ is the test temperature value, *T*_k_՛ is the calculated temperature value, *D*_j_ is the test density value, *D*_j_՛ is the calculated density value, *k* is the sequence number of temperature recording points, *m* is the total number of temperature recording points, *j* is the sequence number of density recording points, and *l* is the total number of density recording points.

In accordance with the aforementioned methodology, the initial fitness value of each particle is calculated. This initial fitness value, denoted as *f*_i_^0^, is assigned as the initial historical optimal fitness value *f*_pi_ for each particle. Among these, the minimum value is selected as the initial historical optimal fitness value *f*_g_ for the group.

(4) The iterative optimization process continues until either the maximum number of iterations is reached or the optimal historical fitness value of the searched population satisfies the predefined accuracy criteria. Upon completion, the parameter set corresponding to the historical optimal fitness of the population represents the inversely derived chemical reaction kinetic parameters. An average of 10 inversion outcomes is considered as the final result. The initial parameter value ranges and the final inversion result are shown in Table 3

## 3. Numerical Simulation of Polyurethane Grout Foaming

In the forward modeling of the polyurethane grout expansion process, the following assumptions are made:

(1) The polyurethane grout is treated as a homogeneous fluid.
(2) The grout maintains uniform mixing, ensuring consistent reaction rates and density throughout.
(3) The reaction vessel is assumed to be thermally insulated.
(4) Heat exchange between the grouting and the surrounding air is neglected.
(5) The generated gas is assumed to completely enter the liquid mixture.

### 3.1. Flow Control Equations

Equation (12) is a mass conservation equation. The density of the expanding polyurethane grout is subject to changes in both its temporal and spatial dimensions. These changes conform to the basic principle of mass conservation, expressed as follows:

(12) $$\frac{\partial \rho }{\partial t}+\frac{\partial (\rho u)}{\partial x}+\frac{\partial (\rho v)}{\partial y}+\frac{\partial (\rho w)}{\partial z}=0$$

where *ρ* is the density, u is the velocity vector, and u, v, and w are the velocity components of the velocity vector u in the x, y, and z directions, respectively.

The grout satisfies the following momentum conservation equation in the expansion process:

(13) $$\left\{\begin{array}{l} \frac{\partial (\rho u)}{\partial t}+\nabla \cdot (\rho uu)=\nabla \cdot (\mu \nabla u)-\frac{\partial p}{\partial x}+S_{u}\\ \frac{\partial (\rho v)}{\partial t}+\nabla \cdot (\rho vu)=\nabla \cdot (\mu \nabla v)-\frac{\partial p}{\partial y}+S_{v}\\ \frac{\partial (\rho w)}{\partial t}+\nabla \cdot (\rho wu)=\nabla \cdot (\mu \nabla w)-\frac{\partial p}{\partial z}+S_{w} \end{array}\right.$$

where t is time, ∇ is the symbol of divergence, p is the pressure on the fluid element, μ is viscosity, and u, v, and w are the components of the velocity vector u in the x, y, and z directions, respectively. $S_{u}$, $S_{v}$, and $S_{w}$ are the source terms in the x, y, and z directions, respectively.

Given the presence of both exothermic reactions and endothermic evaporation, adherence to the energy conservation equation is essential, and it can be represented by the following formula:

(14) $$\frac{\partial }{\partial t}(\rho E^{\prime })+\nabla \cdot [u(\rho E+p)]=\nabla \cdot (\lambda \nabla T)+Q_{E}$$

where the source term $Q_{E}$ of the energy equation is used to describe the heat release of the chemical reaction of the polyurethane polymer grouting and the heat absorption of the physical foaming agent, which represents the heat generation rate of the internal heat source. The formula is as follows:

(15) $$Q_{E}=\alpha_{F}\cdot \frac{\rho_{F}}{\rho_{F}^{0}}\cdot (\Delta H_{\mathrm{OH}}\cdot C_{\mathrm{OH}}^{0}\cdot \frac{dX_{\mathrm{OH}}}{dt}+\Delta H_{W}\cdot C_{W}^{0}\cdot \frac{dX_{W}}{dt}-\Delta h_{V,\mathrm{BA}}\cdot \rho_{F}^{0}\cdot \frac{dr_{\mathrm{BL}}}{dt}$$

where E is energy, E′ is the average energy, λ is the weighted average of thermal conductivity, $\alpha_{F}$ is the volume fraction of polyurethane polymer grouting, $\rho_{F}$ is the polymer density, and $\Delta H_{W}$ and $\Delta H_{\mathrm{OH}}$ are the reaction heats of the foaming reaction and the gel reaction, which are 8.6 × 104 J/g equiv and 7.705 × 10^4^ J/g equiv [27], respectively. $\Delta h_{V,\mathrm{BA}}$ is the endothermic evaporation of the physical foaming agent, and the value is 2.068 × 10^5^ J/kg [28].

In order to determine the concentration of the water and hydroxyl components, as well as the gasification rate of the physical blowing agent, three customized scalar transport equations are formulated as follows [29]:

(16) $$\left\{\begin{array}{l} \frac{\partial }{\partial t}(\rho \alpha_{F}X_{\mathrm{OH}})+\nabla \cdot (\rho \alpha_{F}X_{\mathrm{OH}}u)=\rho \alpha_{F}Q_{\mathrm{Kim},\mathrm{OH}}\\ \frac{\partial }{\partial t}(\rho_{F}\alpha_{F}X_{W})+\nabla \cdot (\rho_{F}\alpha_{F}X_{W}u)=\rho_{F}\alpha_{F}Q_{\mathrm{Kim},W}\\ \frac{\partial }{\partial t}(\rho_{F}\alpha_{F}L_{\mathrm{gas}})+\nabla \cdot (\rho_{F}\alpha_{F}L_{\mathrm{gas}}v)=\rho_{F}\alpha_{F}Q_{\mathrm{BA}} \end{array}\right.$$

where the source terms $Q_{\mathrm{Kin},\mathrm{OH}}$, $Q_{\mathrm{Kim},W}$, and $Q_{\mathrm{BA}}$ represent the conversion of the hydroxyl component and water component and the solubility of the physical blowing agent in polymer grouting with temperature and pressure, respectively. According to the kinetic Equations (4) and (5) and the solubility formula of blowing agent (10), the expression of the source terms $Q_{\mathrm{Kin},\mathrm{OH}}$, $Q_{\mathrm{Kim},W}$, and $Q_{\mathrm{BA}}$ can be obtained, respectively, and expressed as follows:

(17) $$\left\{\begin{array}{l} Q_{\mathrm{Kin},\mathrm{OH}}=A_{OH}\cdot exp(-\frac{E_{OH}}{RT})\cdot c_{OH}^{0}.(1-X_{OH})\cdot (\frac{c_{NCO}^{0}}{c_{OH}^{0}}-2\frac{c_{W}^{0}}{c_{OH}^{0}}X_{W}-X_{OH})\\ Q_{\mathrm{Kin},W}=A_{W}\cdot exp(-\frac{E_{W}}{RT})\cdot c_{OH}^{0}\cdot (1-X_{W})\cdot (\frac{c_{NCO}^{0}}{c_{OH}^{0}}-2\frac{c_{W}^{0}}{c_{OH}^{0}}\cdot X_{W}-X_{OH})\\ Q_{BA}=\left\{\begin{array}{c} \frac{116.9}{700}\cdot \frac{0.01078}{{\left(0.01078T-2.62831\right)}^{2}}\cdot \frac{dT}{dt}\;\;\;\;\;\;\;T\geq T_{\mathrm{BL}}^{0}\\ 0\;\;\;\;\;\;\;\;\;\;\;\;\;\;\;T<T_{\mathrm{BL}}^{0} \end{array}\right. \end{array}\right.$$

Please refer to Appendix A for a comprehensive explanation of the parameters discussed in this paper.

### 3.2. Verification of Numerical Simulation

**Figure 5:**
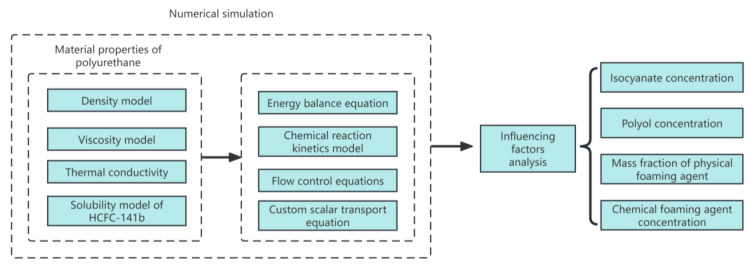
Numerical simulation flow diagram.

**Figure 6:**
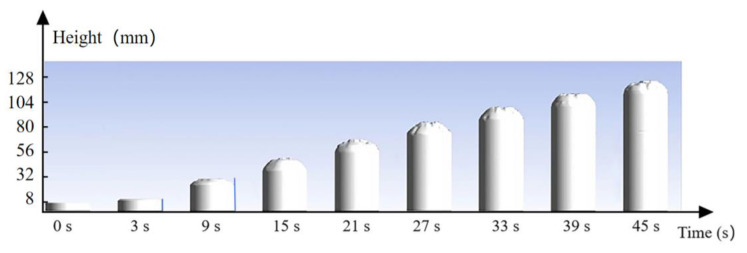
Cloud chart of grout expansion over time.

Figure 5 illustrates the numerical calculation process for the expansion behavior of polyurethane grouting, taking into account the influence of the chemical components. An open cup with filling grout was initially used as the physical model. The upper boundary is defined as the outlet, while the side and bottom surfaces are designated as wall boundaries. The initial volume and density of polyurethane grout are known as V_0_ and ρ_0_, respectively. The finite volume method is employed to discretize the solution domain. For this study, secondary development of the commercial software ANSYS Fluent 2021 R1 was undertaken, which involved incorporating source terms into the energy equations and developing specific transport equations. The governing equations were solved utilizing the SIMPLE algorithm, second-order upwind scheme, and first-order time scheme. Figure 6 illustrates the simulated process of grout expansion under the specified working conditions.

**Figure 7:**
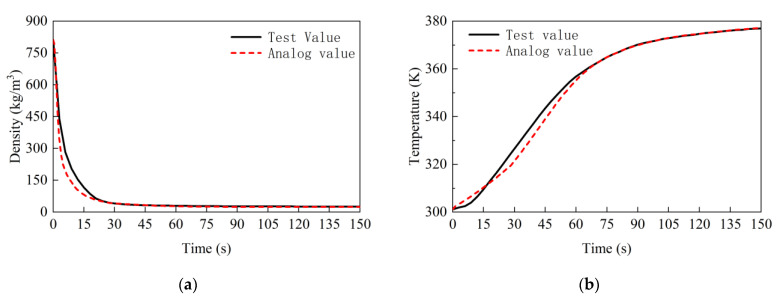
Changes in polymer grouting density and temperature at different times: (**a**) density; (**b**) temperature.

Comparative analysis between the calculated and measured values of the grout density and temperature reveals significant insights. Figure 7 demonstrates that the grouting density diminishes over time, while the temperature increases and eventually stabilizes. It is evident from Figure 7a that the calculated densities are in substantial agreement with the experimental results. The final density calculation yielded 23.87 kg/m^3^, compared to the experimental finding of 25.47 kg/m^3^, indicating a relative error of 6.26%. The temperature at the center of the polyurethane grout was measured and compared to the experimental data, as shown in Figure 7b. The calculated temperature consistently matched the experimental data, with a final calculated temperature of 377.15 K, only 0.33% higher than the highest measured temperature of 376.90 K in the tests. The average relative error between the calculated and experimental results is 2.25%, indicating a strong agreement between them. The validity and applicability of the simulation model are verified.

## 4. Influence of Composition Ratio on Expansion Behavior

In this analysis, the focus was on evaluating the effects of critical components on the expansion characteristics of polymer grouting at room temperature and atmospheric pressure. Variables, such as isocyanate and polyol concentrations, the mass fraction of the physical foaming agent, and the concentration of the chemical foaming agent, were examined. The details of all numerical cases pertaining to this study are presented in Table 4.

### 4.1. Effect of Isocyanate Concentration

**Figure 8:**
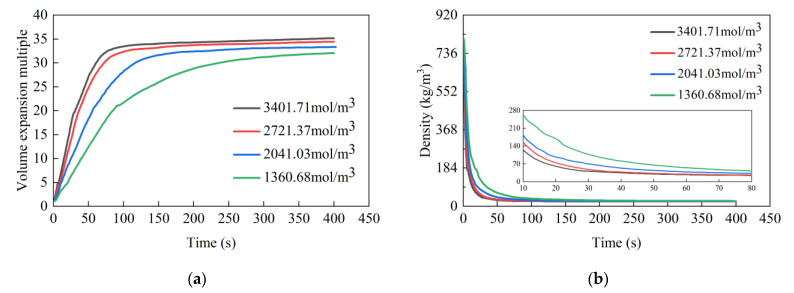
The effect of isocyanate concentration on the expansion multiple and density of high polymer grouting: (**a**) expansion multiple; (**b**) density.

Figure 8 presents the evolution of the volume expansion ratio and density over time under various isocyanate concentration conditions, while Table 5 lists the corresponding final volume expansion ratio and density value. It is evident that the isocyanate concentration exerts a pronounced effect on the expansion ratio of the grout. At isocyanate concentrations of 1360.68, 2041.03, 2721.37, and 3401.71 mol/m^3^, the expansion multiple change rates were, respectively, measured at 0.2276/s, 0.3483/s, 0.5426/s, and 0.6388/s. The resulting final volume expansion multiples were 32.04, 33.36, 34.44, and 35.17, with stabilization occurring at 390 s, 180 s, 165 s, and 150 s, respectively. The latter two conditions exhibited a similar volume expansion rate and final ratio, with the final volume expansion ratio decreasing from an initial 1.32 to 0.73. This trend indicates that an increase in isocyanate concentration led to a higher expansion rate in the grout, an increased final expansion ratio, and a shorter stabilization time. However, a notable observation is the gradually decreasing incremental increase in the grout expansion at a higher isocyanate concentration, implying an upper limit to the effective concentration of isocyanate in the reaction system. When this limit is approached, further increases in isocyanate concentration do not significantly alter the final expansion multiple of the grout volume.

The reason behind this pattern lies in the fact that higher isocyanate concentrations enhance the interaction probability between the chemical foaming agent and isocyanate [29], leading to increased carbon dioxide gas production and heat release, thereby accelerating the expansion rate of the grout and raising the final volume expansion multiple. However, once the isocyanate concentration reaches a saturation point, further increases cease to have a substantial impact on the final volume expansion ratio of the grout.

### 4.2. Effect of Polyol Concentration

**Figure 9:**
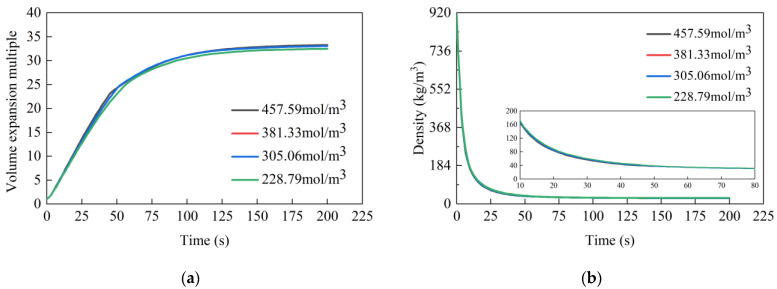
The effect of polyol concentration on the expansion multiple and density of high polymer grouting: (**a**) expansion multiple; (**b**) density.

Figure 9 displays the volume expansion ratio and density of polyurethan grout over time under varying polyol concentrations, with the final values presented in Table 6. The red curve representing 381.33 mol/m^3^ in Figure 9 is covered by other lines because the volume expansion ratio and density of polyurethane slurry at different polyol concentrations are similar. The analysis reveals that under four distinct working conditions, the volume expansion rate, stabilization time, and final expansion multiple of the polyurethan grout are comparatively uniform. Initially, the expansion rate is approximately 0.50/s, decelerating around 50 s, and stabilizing by 130 s, with the final expansion multiple averaging around 33. Overall, the influence of the polyol concentration on the grout expansion process appears to be minimal.

This phenomenon is primarily attributed to the role of polyols in the gel reaction of polyurethane materials. While an increased concentration of polyols can enhance the heat released during the reaction, thereby impacting the total volume change in the grout, it does not lead to additional gas production. Since the volume expansion in the reaction system predominantly arises from the carbon dioxide produced during the foaming reaction and the gasification of the physical foaming agent, variations in polyol concentration do not bring about substantial changes in the volume expansion ratio of the grout.

### 4.3. Effect of the Mass Fraction of the Physical Foaming Agent

**Figure 10:**
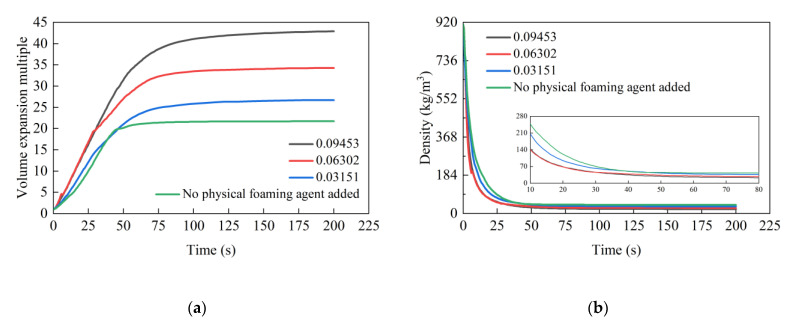
The effect of the physical foaming agent mass fraction on the expansion multiple and density of polymer grouting: (**a**) expansion multiple; (**b**) density.

Figure 10 presents the grout volume expansion ratio and density trends over time under various physical foaming agent mass fractions, with the final results detailed in Table 7. The data clearly indicates that the physical foaming agent significantly affects the grout expansion process. In the absence of the agent, the volume expansion ratio of the grout changes at a rate of around 0.4477/s, achieving a final ratio of 21.71 and stabilizing at about 59 s. When the mass fractions of the physical foaming agent are set at 0.03151, 0.06302, and 0.09453, the expansion rate of the grout changes to 0.4758/s, 0.6437/s, and 0.6544/s, respectively, with final volume expansion multiples of 26.70, 34.14, and 42.87. The increase in the final expansion multiple is directly proportional to the quantity of the physical foaming agent. Stabilization times under these conditions are observed at 78 s, 86 s, and 100 s, respectively. When the mass fraction reaches 0.09453, it approaches the maximum solubility of the physical foaming agent in the mixture at room temperature. Here, the final expansion ratio is about twice that of grout without the agent, and the stabilization time is approximately 1.7 times longer. In comparison, the grout without the physical foaming agent shows a slower expansion rate and a lower final expansion ratio, yet it stabilizes more quickly. An increase in the mass fraction of the agent results in a gradual rise in the expansion rate and final expansion multiple, but with a longer time to reach stabilization.

The behavior can be attributed to the fact that an increased mass fraction of the physical foaming agent leads to a proportional rise in gas production from its gasification, enhancing the volume expansion ratio of the grout. The endothermic gasification process of the physical foaming agent means that larger additions result in more heat absorption, which slows the gasification process and reaction rates, thus delaying the completion of the chemical reaction and the stabilization time of the grout volume.

### 4.4. Effect of the Chemical Foaming Agent Concentration

**Figure 11:**
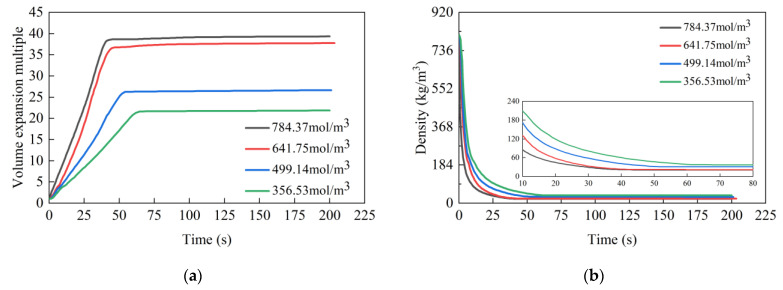
The effect of the concentration of the chemical foaming agent on the expansion ratio and density of high polymer grouting: (**a**) expansion multiple; (**b**) density.

Figure 11 shows the volume expansion ratio and density of grout over time under varying concentration of the chemical foaming agent, with Table 8 detailing the final expansion ratio and densities. The concentration of the chemical foaming agent under four different conditions were 356.53 mol/m^3^, 499.14 mol/m^3^, 641.75 mol/m^3^, and 784.37 mol/m^3^. The corresponding rates of change in the volume expansion rate were 0.333/s, 0.4887/s, 0.8702/s, and 0.9236/s, with final expansion ratios of 21.84, 26.66, 37.75, and 39.32, respectively. The reactions stabilized at 62 s, 54 s, 44 s, and 42 s, respectively. It is observed that as the concentration of the chemical foaming agent increases, there is a corresponding acceleration in the change rate of the grout expansion ratio, an increase in the final expansion ratio, and a decrease in the stabilization time. Similar curves for the two highest concentrations suggest an upper limit to the concentration of the chemical foaming agent. When this threshold is approached, further increases in concentration cease to significantly influence the final expansion ratio of the grout. The volume expansion multiple curve under varying conditions features a notable turning point, marking the moment when the volume change stabilizes. Prior to this point, the grout volume expansion multiple increases approximately linearly with time.

This phenomenon is explained by the fact that increasing the concentration of the chemical foaming agent accelerates the rate of carbon dioxide formation in the foaming reaction, and the increased heat release from reaction hastens the gasification rate of the physical foaming agent. This results in a gradual increase in the rate of change in the expansion ratio. Once the chemical foaming agent concentration reaches a certain level, further increases lead to a diminishing change in the volume expansion ratio of grout [35].

### 4.5. Analysis of the Influence of Various Factors on the Expansion Performance of Polymer Grouting

**Figure 12:**
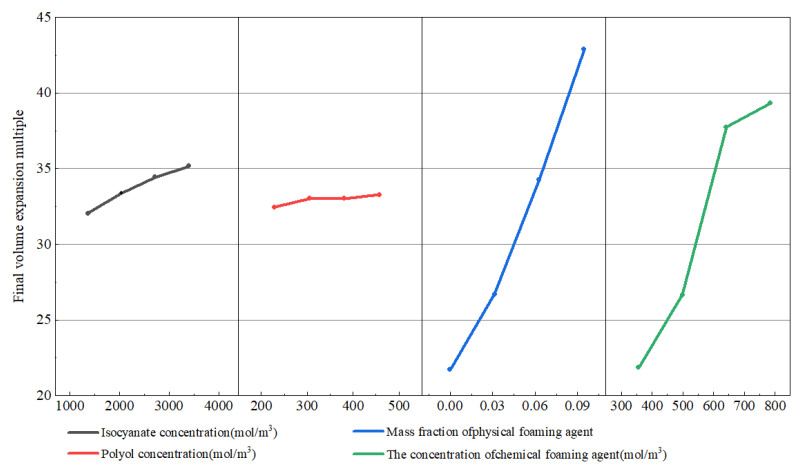
Sensitivity analysis of various factors on the final expansion multiple.

Figure 12 presents the influence of changes in each component concentration on the expansion ratio of the grout under experimental conditions. It clearly shows that the mass fraction of the physical foaming agent and the concentration of the chemical foaming agent have the most pronounced effect on the final volume expansion ratio of the polyurethane grout. The expansion ratios for these components span from 21.71 to 42.87 for the physical foaming agent and 21.84 to 39.32 for the chemical foaming agent, with influence ranges of 21.16 and 17.43, respectively. The isocyanate concentration has a relatively smaller influence, with an expansion ratio range of 32.04 to 35.17 and an influence range of 5.68. The polyols concentration impacts the expansion ratio the least, with the upper limits of volume expansion ratio under the test conditions for each component being 42.87, 33.28, 35.17, and 39.32, respectively.

In order to analyze the sensitivity of the grout expansion ratio to the changes in concentration of different components, we introduced $R_{j-i}$ as the rate of change in the grout volume expansion ratio when the concentration of different components changed. The calculation formula is as follows:

(18) $$R_{j-i}=\left|\frac{S_{j}-S_{i}}{S_{i}}\right|$$

where $R_{j-i}$ is the change rate of the expansion ratio of the grout; $S_{j}$ is the expansion ratio of the grout after the component concentration increases; $S_{i}$ is the expansion ratio of the grout before the component concentration increases.

According to the formula, the sensitivity of the grout expansion ratio to the concentration of various factors is shown in Table 9.

These results indicate that the mass fraction of the physical foaming agent and the concentration of the chemical foaming agent are the most influential factors in determining the final volume expansion ratio of the grout, followed by the isocyanate concentration.

## 5. Conclusions

This study focuses on a self-developed polyurethane polymer material, utilizing particle swarm optimization to identify the chemical reaction kinetic parameters of the grout based on free expansion test results. A chemical reaction model consistent with these results was established. Subsequent simulation analysis under different working conditions explored the impact of the composition ratio of isocyanate, polyol, and physical and chemical foaming agents on the expansion performance of the polyurethane grouting material. The key findings include the following:

(1) The final volume expansion ratio of the polymer grouting increases with increased concentration of isocyanate and the physical and chemical foaming agent within the experimental range. Notably, the mass fraction of the physical foaming agent and the concentration of the chemical foaming agent significantly affect the final expansion ratio. The sensitivity of the expansion ratio to the component proportion was evaluated by analyzing the rate of change in final volume expansion multiple. The sensitivity coefficients for physical and chemical blowing agents were 97.47 and 80.04, respectively, indicating a significant impact. In contrast, the effect of isocyanate was relatively modest, with a sensitivity coefficient of 9.76, while the polyol concentration had minimal impact, with a sensitivity coefficient of 2.53.
(2) The expansion rate of the grout progressively increases with a higher concentration of isocyanate and chemical foaming agent. However, the incremental rise in the final volume expansion ratio diminishes with the same concentration increment. Each of these components has an upper limit concentration. In our study, the upper limits of concentration ratio for isocyanate, polyol, and chemical the blowing agent are 1:0.112:0.231, respectively. Beyond this threshold, there are minimal changes in the final volume expansion ratio.
(3) The volume increase in the grout is directly proportional to the mass fraction increase in the physical foaming agent. The solubility of this agent at a given ambient temperature sets its maximum addition amount and, consequently, its maximal contribution to the volume expansion ratio of the grout. When the mass fraction of the physical foaming agent is 0.09453, the solubility reaches its maximum.

This research provides a preliminary insight into the influence of the composition ratio on the expansion characteristics of the grouting. This was preliminarily studied for self-made polyurethane polymer grouting materials. Future studies will delve into the influence of environmental pressure, temperature, and multi-factor coupling on the expansion process of polyurethane materials, aiming to guide the customization of polyurethane grout with specific expansion performance in geotechnical engineering.

## Figures and Tables

**Table 1:** The ratio and strength of each component in the raw material of polymer grouting.

Formulation	Raw Material	Source	Mass (%)	Strength
Isocyanate	MDI-50	Wanhua, Yantai, China	46.9	Enhancing the flexibility and elongation.
Polyols	Polyether N220		43.6	Reacting with diisocyanates, resulting in flexible, hydrolysis-resistant, heat-resistant linear polyurethanes.
Foaming agent	Physical foaming agent HCFC-141b		6.2	Low thermal conductivity in gases and highly compatible with polyols and isocyanates.
	Chemical foaming agent water		1.4	Low cost, non-toxic, non-flammable, and no adverse effects on the ozone layer.
Catalyst	Catalytic agent A33		1.4	Causing the initial viscosity increasing rapidly, enhancing the stability and flexibility.
Foam stabilizers	Foam stabilizer AK8806		0.5	Good defoaming and foaming function and excellent thermal stability.

**Table 2:** Particle swarm algorithm input value setting.

Parameters			Value	Unit
Particle swarm parameter	n	The population size	60	/
	t	The iteration count	50	/
	$c_{1}$	Learning factors	0.5	/
	$c_{2}$		0.5	/
Material parameter	$C_{OH}^{0}$	The initial concentration of the hydroxyl	381.33	mol/m^3^
	$C_{\mathrm{NCO}}^{0}$	The initial concentration of the isocyanate	3401.71	mol/m^3^
	$C_{W}^{0}$	The initial concentration of the water component	713.06	mol/m^3^
	$r_{\mathrm{BL}}^{0}$	The initial mass fraction of the physical foaming agent	0.06726	/
	$\rho_{P}^{0}$	The initial density of the grout	893.49	kg/m^3^
	$T_{0}$	The initial temperature	301.15	K

**Table 3:** Inversion results of the chemical reaction kinetics parameters.

Kinetic Parameters	The Range of the Initial Value		Inversion Parameter Values	Unit
	Minimum	Maximum		
$A_{W}$	20.996	30.776	25.571	m^3^/g equiv/s
$E_{W}$	35,810	40,120	38,998.51	J/g mol
$A_{\mathrm{OH}}$	2.0348	3.0048	2.471	m^3^/g equiv/s
$E_{OH}$	35,400	39,400	37,015.27	J/g mol

**Table 4:** Numerical cases.

Serial Number	Variable	$C_{NCO}^{0}$/(mol/m^3^)	$C_{OH}^{0}$/(mol/m^3^)	$r_{BL}^{0}$	$C_{W}^{0}$/(mol/m^3^)	$C_{NCO}^{0}$ $:C_{OH}^{0}$ $:C_{W}^{0}$
1	Initial condition	3401.71	381.33	0.06302	713.06	1:0.112:0.21
2	$C_{NCO}^{0}$	1360.68	381.33	0.06302	713.06	0.4:0.112:0.21
3		2041.03				0.6:0.112:0.21
4		2721.37				0.8:0.112:0.21
5	$C_{OH}^{0}$	3401.71	229.39	0.06302	713.06	1:0.067:0.21
6			305.06			1:0.090:0.21
7			457.59			1:0.135:0.21
8	$r_{BL}^{0}$	3401.71	381.33	0	713.06	1:0.112:0.21
9				0.03151		
10				0.09453		
11	$C_{W}^{0}$	3401.71	381.33	0.06302	356.53	1:0.112:0.105
12					499.14	1:0.112:0.147
13					641.75	1:0.112:0.189
14					784.37	1:0.112:0.231

**Table 5:** The final volume expansion multiple and final density of the grout at different isocyanate concentrations.

Serial Number	1	2	3	4
$C_{NCO}^{0}$/(mol/m^3^)	1360.68	2041.03	2721.37	3401.71
Final volume expansion multiple	32.04	33.36	34.44	35.17
Final density/(kg/m^3^)	25.32	24.34	23.58	23.07
Increase in final volume expansion multiple	/	1.32	1.08	0.73

**Table 6:** The final volume expansion multiple and final density of the grouting under different polyol concentrations.

Serial Number	1	2	3	4
$C_{OH}^{0}$/(mol/m^3^)	228.79	305.06	381.33	457.59
Final volume expansion multiple	32.46	33.02	33.02	33.28
Final density/(kg/m^3^)	28.01	27.53	27.53	27.32

**Table 7:** The final value of the volume expansion multiple and stabilizing time of the grouting under different mass fractions of the physical foaming agent.

Serial Number	1	2	3	4
$r_{BL}^{0}$	0	0.03151	0.06302	0.09453
Final volume expansion multiple	21.71	26.70	34.28	42.87
Increase in final volume expansion multiple	/	4.99	7.58	8.59
Stabilizing time/s	59	78	86	100

**Table 8:** The final volume expansion multiple and density of the grouting at the concentration of the chemical foaming agent.

Serial Number	1	2	3	4
$C_{W}^{0}$/(mol/m^3^)	356.53	499.14	641.75	784.37
Final volume expansion multiple	21.84	26.66	37.75	39.32
Final density/(kg/m^3^)	37.12	30.42	21.49	20.66
Increase in final volume expansion multiple	/	4.82	11.09	1.57
Reduction in final density/(kg/m^3^)	/	6.70	8.93	0.83

**Table 9:** Grout expansion ratio sensitivity factor to the component.

Variable	The Changes in the Component Concentration	Unit	$R_{j-i}$ (%)
$C_{NCO}^{0}$	1360.68→2041.03	mol/m^3^	4.12
	2041.03→2721.37		3.24
	2721.37→3401.71		2.10
	1360.68→3401.71		9.76
$C_{OH}^{0}$	229.39→305.06	mol/m^3^	1.73
	305.06→381.33		/
	381.33→457.59		0.79
	229.39→457.59		2.53
$r_{BL}^{0}$	0→0.03151	/	22.98
	0.03151→0.06302		28.39
	0.06302→0.09453		25.06
	0→0.09453		97.47
$C_{W}^{0}$	356.53→499.14	mol/m^3^	22.07
	499.14→641.75		41.60
	641.75→784.37		4.16
	356.53→784.37		80.04

## Data Availability

Data are contained within the article.

## Appendix A

**Table A1:** Formula symbol identification.

Formula	Parameter	Definition
The kinetic equations	$X_{\mathrm{OH}}$	The conversion rate of the hydroxyl component
	$A_{\mathrm{OH}}$	The pre-exponential factor of the gel reaction
	$E_{\mathrm{OH}}$	The activation energy of the gel reaction
	R	The ideal gas constant
	T	The current temperature
	$C_{{\;\;}_{\mathrm{OH}}}^{0}$	The molar concentration of the hydroxyl component at the initial time
	$C_{{\;\;}_{\mathrm{NCO}}}^{0}$	The molar concentration of the isocyanate component at the initial time
	$C_{{\;\;}_{W}}^{0}$	The initial molar concentration of the water component
	$X_{W}$	The conversion rate of the water component
	$A_{W}$	The pre-exponential factor of the foaming reaction
	$E_{W}$	The activation energy of the foaming reaction
Energy balance equation	$C_{P}$	The heat capacity of the polymer grouting
	$C_{{\mathrm{CO}}_{2}}$	The heat capacity of carbon dioxide
	$C_{W}$	The heat capacity of water
	$C_{\mathrm{BG}}$	The heat capacity of the gaseous physical foaming agent
	$C_{\mathrm{BL}}$	The heat capacity of the liquid physical foaming agent
	$r_{{\mathrm{CO}}_{2}}$	The mass fraction of carbon dioxide
	$r_{W}$	The mass fraction of water
	$r_{\mathrm{BG}}$	The mass fraction of the gaseous physical foaming agent
	$\Delta H_{W}$	The heat of the foaming reaction
	λ	The heat of the evaporation and absorption of the physical foaming agent
Material properties of polyurethane	$r_{{\;\;}_{W}}^{0}$	The initial mass fraction of water
	$r_{{\;\;}_{\mathrm{BL}}}^{0}$	The initial mass fraction of the physical foaming agent
	$R_{g}$	The ideal gas constant
	$r_{\mathrm{BG}}$	The mass fraction of the gaseous physical foaming agent
	$r_{\mathrm{BL}}$	The mass fraction of the liquid physical foaming agent
	$r_{W}$	The mass fraction of water
	$M_{W}$	The molar mass of water
	$M_{B}$	The molar mass of the physical foaming agent
	$\rho_{\mathrm{BL}}$	The density of liquid physical foaming agent
	$\rho_{W}$	The density of water
	$\rho_{P}$	The density of the polymer mixture
	$X_{\mathrm{NCO},\mathrm{gel}}$	The conversion rate of the isocyanate gel
	$\lambda_{F}$	The thermal conductivity of the grout
	$\rho_{F}$	The density of the grout
The particle swarm optimization algorithm	n	The population size
	d	The particle search space range and dimension
	$c_{1}$	Learning factors
	$c_{2}$	
	t	The iteration count
	ε	The convergence accuracy
	$\rho_{{\;\;}_{P}}^{0}$	The initial density of the grout
	$T_{0}$	The initial temperature
	T_k_	The test temperature value
	T_k_′	The calculated temperature value
	D_j_	The test density value
	D_j_′	The calculated density value
Flow control equations	ρ	The density
	***u***	The velocity vector
	*u*, *v*, *w*	The velocity components of velocity vector ***u*** in the *x*, *y*, and *z* directions
	t	Time
	∇	The symbol of divergence
	p	The pressure on the fluid element
	E	Energy
	E′	The average energy
	λ	The weighted average of thermal conductivity
	$\alpha_{F}$	The volume fraction of the polyurethane polymer grout
	$\Delta h_{V,\mathrm{BA}}$	The endothermic evaporation of the physical foaming agent
